# “Probe-ing” Deeper: Ultrasound-Assisted Lumbar Puncture Training in Neurology

**DOI:** 10.1212/NE9.0000000000200368

**Published:** 2026-10-01

**Authors:** John H. Erdman, Daniel S. Harrison

**Affiliations:** 1 Department of Neurology, Boston University School of Medicine, MA; and; 2 Department of Neurology, Boston Medical Center, MA.

Competence in use of point-of-care ultrasound (POCUS) as both a diagnostic and procedural tool is highlighted as a graduation goal in the Accreditation Council for Graduate Medical Education (ACGME) milestones for a variety of medical specialties. Despite growing efforts to improve training and potential cranial, cerebrovascular, ocular, and neuromuscular applications, point-of-care ultrasound is only moderately used among some groups of neurology clinicians.^1^ Ultrasound-assisted lumbar puncture (USLP) and ultrasound guidance for similar neuraxial procedures have been evaluated for safety and efficacy in other specialties (most notably emergency medicine and anesthesiology). However, data supporting the use of this technique by neurologists and neurology trainees are limited.

Grubman et al.^2^ review the possible benefits of USLP in neurology, analyze previous studies in other fields, and describe future steps for more widespread adoption of the technique in their narrative review. The authors begin by summarizing data on traditional, landmark-based LP failure rates, citing a range of 7.7%–28% when performed by neurologists. A prior systematic review and meta-analysis of USLP suggested that the failure rate when using this technique is approximately 10%.^3^ One reason to consider further study of USLP in the hands of neurologists is that, if our landmark-guided success rates are higher than those of our colleagues, there may be less to gain for procedural success rates and safety when applied across all patient populations in our context. If, therefore, the benefits of ultrasound assistance when compared with landmark guidance are marginal, then the educational costs (including incorporating USLP into the ACGME neurology milestones and developing and implementing USLP curricula) and financial investment (for example, purchase of ultrasound machines for neurology departments and practices that may not otherwise require them) may outweigh the benefits. Future work evaluating this cost-benefit analysis in our unique context could be of value.

It is clear, however, that when studied in other specialties, USLP has resulted in increases in overall and first-pass success rates, with reductions in patient-reported pain scores and possibly rates of “traumatic” LPs. The authors further emphasize that the benefits of USLP may be even more pronounced in patients who are overweight or have “difficult” anatomy. For example, in 1 randomized controlled study of 40 patients who were overweight, had scoliosis, or had prior lumbar spinal surgery, patients randomized to USLP had significantly higher first-pass success rates (80% vs 35%, *p* = 0.009, number needed to treat = 2.22) and faster procedure times.^4^ These findings were consistent with the results of a larger prospective study of USLP in patients who were overweight after controlling for “back disease” (including lumbar scoliosis, L3-S1 spinal stenosis with or without surgery, lumbar arthritis, and lumbar spondylosis).^5^ Considering these data, perhaps patients who seem most likely to benefit (for example, those with the highest body mass indices [BMIs] or severe back disease) should always have LP performed with ultrasound assistance, regardless of the discipline of the specialist performing the procedure. Additional research seeking to clarify an optimal BMI cut point or specific anatomic features that identify patients who may benefit most from USLP could inform future practice.

As the authors highlight, competency-based training, which could be designed using Sawyer's framework, ensures that competence is developed and maintained among those learning new procedures, such as USLP.^6^ Another competency-based approach, simulation-based mastery learning, has previously been shown to improve landmark-guided LP performance among internal medicine residents.^7^ Although time and resource intensive, neurology educators inspired by the successes of USLP in other specialties who are ready for direct implementation in their learners should lean on these evidence-based teaching approaches, as opposed to brief bedside or simulation training, which may have limited the efficacy of USLP in some prior trials.^8,9^ Partnering with anesthesiologists and emergency medicine colleagues who may have more experience with and expertise in USLP is another strategy that may be effective in the early training and implementation of USLP in neurology, while neurology trainees and practicing neurologists alike seek to develop our own competence in performing this procedure.

Just as implementation of USLP training and practice in neurology will require rigorous, evidence-based teaching strategies to be successful, future research in this space will have to be of very high quality to add to the existing body of literature. Interventions such as USLP supported by systematic reviews and meta-analyses of randomized controlled trials with patient outcome data are uncommon in neurology education.^10^ Controlled, multicenter studies focusing on higher level outcomes such as trainee performance, patient outcomes, and health care or implementation costs may be effective in showing that the successes of USLP in other specialties translate to neurology.

Although it is unclear that data from other specialties supports the cost-effectiveness and clinical benefit of a widespread “ultrasound-assisted first” approach for all LPs performed by neurology clinicians, it is plausible that implementation of USLP in neurology could benefit the subsection of our patients with the most challenging anatomy and urgent need for diagnostic clarity. We know which competency-based educational tools can be leveraged to maximize the chances of successful implementation in neurology. Empowering neurologists and neurology trainees to perform LPs at bedside in the interest of swift diagnosis and treatment is a worthwhile goal. Are further study and implementation of USLP in our context a pragmatic means to these ends? “Probe-ably” so.
